# Understanding the vaccine stance of Italian tweets and addressing language changes through the COVID-19 pandemic: Development and validation of a machine learning model

**DOI:** 10.3389/fpubh.2022.948880

**Published:** 2022-07-29

**Authors:** Susan Cheatham, Per E. Kummervold, Lorenza Parisi, Barbara Lanfranchi, Ileana Croci, Francesca Comunello, Maria Cristina Rota, Antonietta Filia, Alberto Eugenio Tozzi, Caterina Rizzo, Francesco Gesualdo

**Affiliations:** ^1^Multifactorial and Complex Diseases Research Area, Bambino Gesù Children's Hospital, IRCCS, Rome, Italy; ^2^Vaccine Research Department, FISABIO-Public Health, Valencia, Spain; ^3^Department of Human Sciences, Link Campus University, Rome, Italy; ^4^Department of Communication and Social Research, Sapienza University, Rome, Italy; ^5^Department of Infectious Diseases, Istituto Superiore di Sanità, Rome, Italy; ^6^Department of Translational Research and New Technologies in Medicine and Surgery, Pisa University, Pisa, Italy

**Keywords:** vaccines, machine learning, artificial intelligence, vaccination hesitancy, Transformer model

## Abstract

Social media is increasingly being used to express opinions and attitudes toward vaccines. The vaccine stance of social media posts can be classified in almost real-time using machine learning. We describe the use of a Transformer-based machine learning model for analyzing vaccine stance of Italian tweets, and demonstrate the need to address changes over time in vaccine-related language, through periodic model retraining. Vaccine-related tweets were collected through a platform developed for the European Joint Action on Vaccination. Two datasets were collected, the first between November 2019 and June 2020, the second from April to September 2021. The tweets were manually categorized by three independent annotators. After cleaning, the total dataset consisted of 1,736 tweets with 3 categories (promotional, neutral, and discouraging). The manually classified tweets were used to train and test various machine learning models. The model that classified the data most similarly to humans was XLM-Roberta-large, a multilingual version of the Transformer-based model RoBERTa. The model hyper-parameters were tuned and then the model ran five times. The fine-tuned model with the best F-score over the validation dataset was selected. Running the selected fine-tuned model on just the first test dataset resulted in an accuracy of 72.8% (F-score 0.713). Using this model on the second test dataset resulted in a 10% drop in accuracy to 62.1% (F-score 0.617), indicating that the model recognized a difference in language between the datasets. On the combined test datasets the accuracy was 70.1% (F-score 0.689). Retraining the model using data from the first and second datasets increased the accuracy over the second test dataset to 71.3% (F-score 0.713), a 9% improvement from when using just the first dataset for training. The accuracy over the first test dataset remained the same at 72.8% (F-score 0.721). The accuracy over the combined test datasets was then 72.4% (F-score 0.720), a 2% improvement. Through fine-tuning a machine-learning model on task-specific data, the accuracy achieved in categorizing tweets was close to that expected by a single human annotator. Regular training of machine-learning models with recent data is advisable to maximize accuracy.

## Introduction

In 2019, the World Health Organization listed vaccine hesitancy (1) among the top ten threats to global health (2). Decreased confidence in vaccines can have a serious impact on vaccine uptake, impairing vaccination programs and ultimately leading to a re-emergence of vaccine preventable diseases (3, 4). In the context of the COVID-19 pandemic, the impact of vaccine hesitancy has been evident. Lack of confidence for and actual refusal of COVID-19 vaccines has been ubiquitous, especially in high-income countries (5). A higher morbidity and mortality by COVID-19 has been reported in unvaccinated than in vaccinated individuals (6, 7), and limited vaccine uptake could also reduce the possibility of relaxing non-pharmaceutical interventions in the population (8).

Monitoring vaccine confidence and its determinants, which are typically context-specific, is crucial to understanding the reasons behind willingness to be vaccinated and to inform actions aimed at restoring or maintaining confidence in vaccines.

Social media is increasingly being used to express opinions and attitudes toward vaccines. Social media enables an unmediated circulation of information, which has facilitated the diffusion of misinformation and disinformation, both at the regional-national level and across countries (9), and decreased trust toward official sources of health information (10).

The role of social media in vaccine communication has been often studied, including investigations on the use of social media as a source of information on vaccines (11) and as means to deliver vaccine promotion campaigns (12). Researchers have also analyzed findings from social media monitoring projects (13, 14). Social media monitoring has been recommended as a means to complement classical qualitative research on vaccine hesitancy (15, 16), and to integrate classic vaccine-preventable disease surveillance (17), due to its potential to provide health authorities in real time with large sets of data, spontaneously provided by social media users.

The overabundance of information circulating on the web, both from reliable and from questionable sources, has been defined by the World Health Organization as the “infodemic” (18). Recently, the WHO developed a framework to effectively respond to the infodemic related to COVID-19, including a recommendation to “understand the circulating narratives and changes in the flow of information, questions, and misinformation in communities” and to systematically apply analysis of online conversation (19).

An accurate analysis of social media posts requires an extensive amount of time and resources, which might negatively impact the potential timeliness of social media monitoring compared to classical methods to assess vaccine confidence. Semi-automated analytical techniques have been used to overcome this limit, and different methodologies for natural language processing (NLP) have been applied to this aim (20).

The field of NLP has seen many advances in the last 30 years, moving from grammar-like rules to analyzing neighboring words, representing words as vectors (Word2Vec) (21, 22) and word embeddings (GloVe) (23). Deep neural nets were developed based on the human brain framework, where processing layers or neurons connect together to get from an input to a result (24, 25). In 2015 the attention mechanism was proposed which was a huge success and led Google to propose a new network architecture known as the Transformer (26). Bidirectional Encoder Representations from Transformers (BERT) models were a paradigm shift in NLP.

Sentiment analysis is frequently used in studying public opinion (social media marketing, opinion mining, results of media campaigns, brand reputation, etc.). Sentiment analysis classifies the overall tone or language of a text, whether it is positive, negative or neutral, without considering the meaning or message of the text (27). For this reason, sentiment analysis may not be the most appropriate method for analyzing vaccine conversations on social media: a tweet criticizing the anti-vax movement can have a negative sentiment, despite expressing a favorable opinion toward vaccines. Recently, stance analysis has been proposed as an alternative method to study vaccine-related social media posts, as this type of analysis does not take into account the tone of the text but rather determines favorability toward a chosen topic of interest (28). Kummervold et al. used stance analysis to explore the discourse on vaccination during pregnancy, reaching an accuracy of 81.8% in stance classification (29).

One of the biggest challenges in NLP is the shortage of training data. Modern deep learning-based NLP models benefit from large amounts of data, using millions, or even billions of annotated training examples. However, most task-specific datasets contain only a few hundred or perhaps thousand human-labeled training examples. To help close this gap in data, a variety of techniques for training general purpose language representation models have been developed, including using enormous amounts of unannotated text from the web to pre-train a model. The pre-trained model can then be fine-tuned on small task-specific datasets, resulting in substantial accuracy improvements compared to training on just the small task-specific datasets.

One additional, critical issue that may impair the precision of automatic text analysis for health or vaccine-related contents is the changes that commonly occur in the everyday language used during health emergencies (30). The language used by most social media users in conversations regarding vaccines has deeply changed since the first phase of the pandemic, when initial data on COVID-19 vaccine studies started to circulate, and even more so since the available COVID-19 vaccines were rolled-out across the globe. The impact of these changes on the performance of NLP models for automatic stance classification has not been investigated before.

In this study, we describe the development, fine-tuning and testing of a NLP machine learning model aimed at automatically classifying the stance of vaccine related tweets in Italian. Moreover, we analyze the impact of the change of vaccine-related language during the pandemic on the model performance.

The present project has been conducted by the Bambino Gesù Children's Hospital in the context of the European Joint Action on Vaccination (EU-JAV) (31). EU-JAV is a European project that aims at spurring long-lasting European cooperation against vaccine-preventable diseases. In the context of the EU-JAV's Work Package 8, the Italian National Institute of Health, in collaboration with the Bambino Gesù Children's Hospital, developed a web platform to monitor vaccination discourse on the web and on social media, in particular on Twitter.

## Methods

The development of the NLP machine learning models for vaccine-stance classification was based on an annotated database of vaccine-related tweets in Italian, posted between November 2019 and September 2021 (see below for details). Based on a qualitative exploration of tweets, a substantial change in the language was observed by the researchers before and after the COVID-19 vaccine was rolled out. Inspired by this observation, we decided to study the impact of the change in the vaccine-related discourse through the pandemic on the performance of the model. Our hypothesis was that, if the model was just trained on tweets posted before the COVID-19 vaccine roll-out, and its performance dropped when testing on tweets posted after the roll-out, then this might indicate a change of language and a need for regular model training. Thus, first, we used pre-roll-out tweets for training, validation and testing. Secondly, we tested the model on post-roll-out tweets and the combined datasets. Finally, we used the combined datasets for training and validation, and we tested the model on pre-roll-out tweets, post-roll-out tweets and the combined datasets.

### Data sources

The EU-JAV platform for social media monitoring has been collecting tweets through the Twitter API (standard v1.1) in Italian, French and Spanish since November 2019. Vaccine-related tweets in Italian were downloaded using the following search filter, validated through a structured framework (32) (Vaccino OR Vaccini OR Vaccinazione OR Vaccinazioni OR Vaccinato OR Vaccinata OR Vaccinati OR Vaccinate OR Immunizzazione OR Immunizzato OR Immunizzata OR Immunizzati OR Immunizzate OR Novax).

From the platform's database, we downloaded a random sample of 3,000 tweets in Italian, published between November 2019 and June 2020 (dataset A). Subsequently, we extracted an additional sample of 800 vaccine-related tweets in Italian published between April 2021 and September 2021 (dataset B).

### Transformer models and tokenization

Cognitive attention allows us to concentrate on selected stimuli. It is not just about centering focus on one particular thing; it also involves ignoring a great deal of competing information and stimuli. In machine learning, attention is a technique that mimics cognitive attention. The effect enhances the important parts of the input data and fades out the rest. Which part of the data is more important depends on the context and is learned through training.

A Transformer is a machine learning model that adopts the technique of attention, weighing the significance of each part of the input data. Attention is important in language since context is essential to assigning the meaning of a word in a sentence.

BERT is a Transformer-based machine learning technique for NLP (26). Text is analyzed as a complete sequence of words. The Transformer weighs the importance of different parts of the text and identifies the context that confers meaning to a word in a sentence. Models have different sizes dependent upon the numbers of encoder layers. BERT-base models have 12 layers whereas BERT-large models have 24. The basic principle of deep learning is based on mimicking the operation of neurons in the human brain such that multiple layers are stacked up.

Training deep learning NLP models from scratch is costly, time-consuming, and requires massive amounts of data. Transfer learning techniques customize existing pre-trained models to suit a specific NLP task. Relatively small amounts of data are sufficient for such fine-tuning.

When published in late 2018, BERT demonstrated state-of-the-art results. Since then, various BERT based models have arrived on the scene. Different models vary slightly, but in general, all pre-training is done in a fully unsupervised manner. Unsupervised learning uses unlabeled data, the model decides for itself the patterns within the data. This process generates a general language model that is used as input for supervised fine-tuning of specific language processing tasks. Supervised fine-tuning requires labeled data.

Twitter is a popular microblogging platform where people often express their opinions implicitly or explicitly. Twitter data (tweets) is a rich source of information on a large set of topics. Data can be extracted related to specific keywords, to study trends in the social conversation. Annotating, or labeling data, is a very time consuming task, thus it is difficult to acquire a large dataset of labeled data. Well labeled data is key to the performance of the machine learning model. Labeled data allows a model to be trained to understand the nuances of the subject and the language used in a specific use-case. The more data available, the more examples the model has to understand which category to place a tweet.

Machine learning models cannot work directly on text. Text needs to be separated into smaller units (tokens), then converted to numbers (vectors). This important processing step is carried out by a tool called a “tokenizer”. For example, “coronavirus: modern testing” could be tokenized as “‘_corona’, ‘virus’, ‘:’, ‘_modern’, ‘_testing”’, which is then converted to numbers: [0, 109728, 76912, 12, 5744, 134234, 63, 2]. When text is tokenized, links and usernames can become so split up that they are not useful to the model and can even impair the performance of the model. For example, the link https://t.co/Ub5NJddOYm could be tokenized as “_https”, “://”, “t”, “.”, “co”, “/”, “U”, “b”, “5”, “NJ”, “dd”, “OY”, “m” which is not very useful. A token within a link or username might inadvertently be a token the model associates with a certain stance, even though a link or username does not in itself confer a stance (see Supplementary Table 1 for definitions of Machine learning terminology).

### Data cleaning

Prior to the use of the data by the machine learning models:

- Non-relevant tweets were removed (i.e., Tweets not in Italian, or those using the word “vaccine” in a figurative way).
- Tweets that were replies, and thus not clear in their stance without following the conversation, were identified and removed.

Subsequently, a Python script cleaned the data:

- The “RT” retweet tag was removed.
- Duplicates were identified using a Levenshtein distance <22. The Levenshtein distance is the minimum number of single-character edits (insertions, deletions or substitutions) required to change one sequence of text into another. For shorter tweets, of <130 characters, this distance was scaled, since more edits per character could result in falsely identifying duplicates. The distance was tuned using the data, to ensure correct identification of duplicates.
- Tweets with <5 words were removed since, in most cases, it was not possible to discern stance from such limited information.
- Text was converted to lowercase.
- All usernames and links were replaced with a common tag (“user” or “link,” respectively).

The cleaned datasets included 1,378 (dataset A) and 526 (dataset B) tweets. The number of tweets remaining after each cleaning step are shown in Supplementary Table 2.

### Manual labeling

Each tweet was analyzed by 3 independent human annotators and labeled as promotional, neutral, discouraging, ambiguous or indiscernible, based on the stance toward vaccines expressed by the tweet's author, as in Martin 2020 and Kummervold 2021. In case of discrepancies, a univocal classification, upon which all coders agreed, was reached after collegial discussion among the coders. The 4 stance categories are defined in Table 1.

**Table 1:** Stance categories.

**Category**	**Description**	**Twitter example—Italian (original)**	**Twitter example—English translation**
Promotional	Communicate public health benefits or safety of vaccinations, encourage vaccination, describe risks of not vaccinating or refute claims that vaccines are dangerous. Include tweets clearly criticizing anti-vax persons.	I miei figli sono vaccinati per la #varicella.C'è stata una bella epidemia e loro stanno bene, felici e senza aver preso nulla. Davide è del 2012 e abbiamo pagato il vaccino perché era a pagamento. Rinunciate a qualsiasi cosa ma #vaccinate per i vostri figli e per gli altri.	My children are vaccinated against #chickenpox. There's been an epidemic in their school and they've been fine, happy, did not have any symptom. Davide was born in 2012, we payed for the vaccine because it was not free for his age. Renounce everything but #vaccinate, for your children and for the others.
Neutral	Often statements, including factual recommendations about vaccines. Contain no elements of uncertainty, promotional or negative content regarding vaccines. Doubts about political decisions regarding vaccination programs may be expressed, but vaccines are referred to in a neutral way.	Coronavirus, Oms: Per vaccino dovremo attendere almeno 18 mesi	Coronavirus, WHO: For the vaccine we'll have to wait at least for 18 months.
Discouraging	Contain negative attitudes/arguments against vaccines. Contain questions regarding effectiveness/safety or possibility of adverse reactions that may or may not be proven. Discourage the use of recommended vaccines.	#vaccini INVALIDA per colpa di un vaccino #antinfluenzale. Il ministero della Salute condannato a risarcirla. I vaccini sono FARMACI. Diffidate da tutti quelli che vi dicono che sono sicuri e che non hanno #effetticollaterali. Indietro non si torna.	#vaccines DISABLED for a #fluvaccine. Ministry of Health condemned to pay. Vaccines are DRUGS. Be wary of all those that say that vaccines are safe and do not have #sideffects. You can't go back.
Ambiguous	Contain indecision or uncertainty on the risks or benefits of vaccination.	Raga io devo ancora decidere ed ho paura...se mi vaccino non so gli effetti collaterali....se non mi vaccino e mi prendo il Covid?poi x quanto sarò in pericolo?se mi vaccino x quanto funzionera'?ho paura...consigli???	I still have to decide and I'm scared…if I get the vaccine, I don't know side effects…but what if I don't get the vaccine and I get covid? for how long will I be in danger? If I get vaccinated, how long is the vaccine going to work? I'm scared…any advice?

In addition, we labeled as indiscernible those tweets in which the stance could not be understood, e.g., tweets including sarcasm or using an ambiguous language.

**Figure 1:**
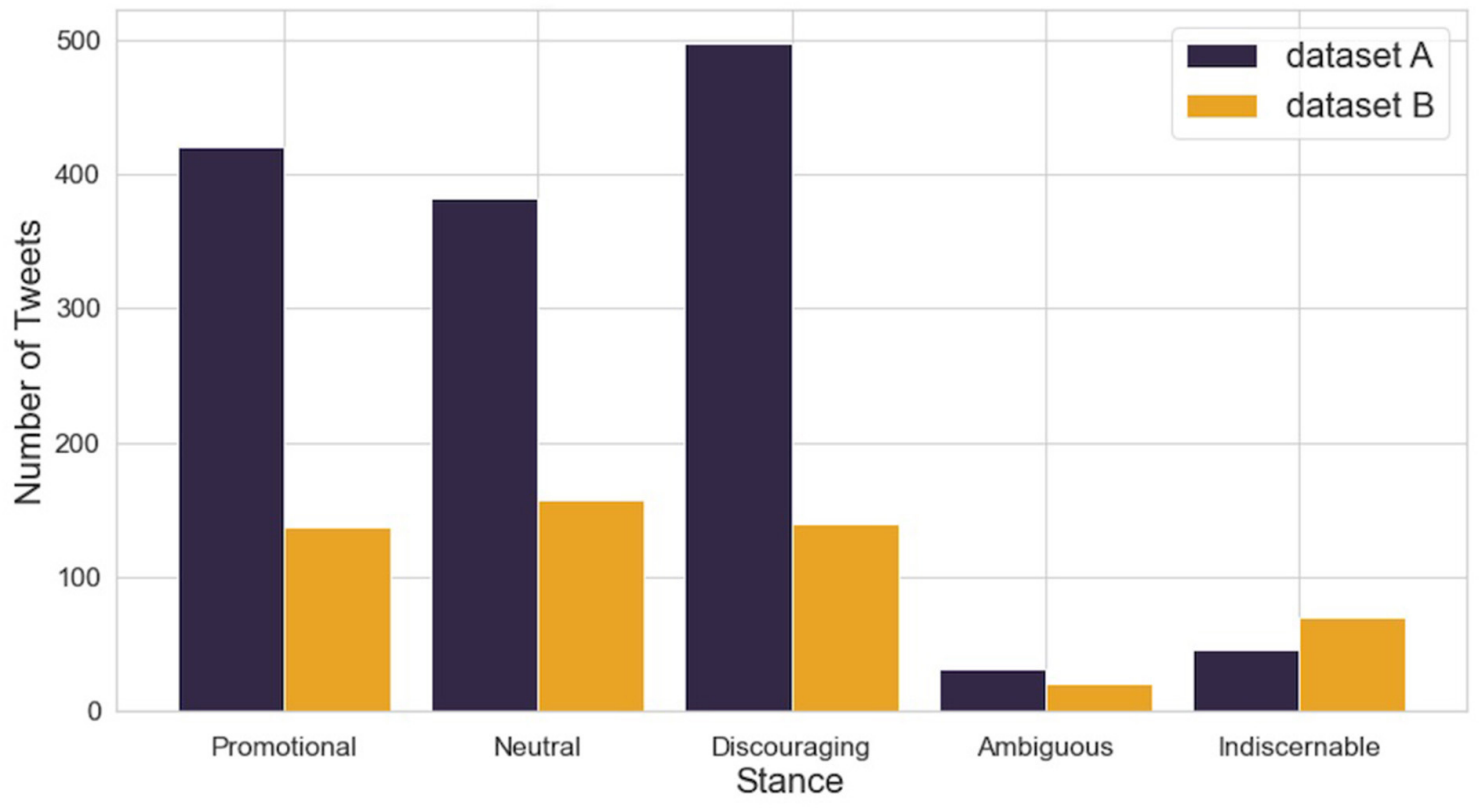
Dataset stance labels after cleaning.

Given the very low proportion of ambiguous and indiscernible tweets (see Figure 1), and the inconsistent nature of the texts, the tweets belonging to these two categories were removed and the data was reduced to just 3 categories: promotional, neutral and discouraging. This action was also justified by running the model over the full dataset with 5 categories: the model then classified the majority of the ambiguous or indiscernible tweets as promotional, neutral or ambiguous, thus indicating that just three categories was more appropriate (Supplementary Figure 1). Three categories also gives the advantage of similar numbers of data within each category, which is helpful for the machine learning models. The final dataset consisted of 1,736 unique tweets, with dataset A containing 1,301 tweets and dataset B containing 435 tweets, as shown in Table 2.

**Table 2:** Dataset after cleaning, 3 categories.

**Stance**	**Dataset A**	**Dataset**	**Dataset B**	**Dataset**	**Total number of**	**Percentage of**
	**Number of tweets**	**A %**	**Number of tweets**	**B %**	**unique tweets**	**total tweets (%)**
Promotional	421	32.4	137	31.5	558	31.5
Neutral	382	29.3	158	36.3	540	30.5
Discouraging	498	38.3	140	32,2	638	38.0
Total	1,301	74.9	435	25.1	1,736	100

Considering just 3 categories (promotional, neutral, discouraging), the accuracies for the individual annotators compared to the final agreed score were 79.2, 83.9 and 88.1%. Of this data, 62.6% of the tweets were coded identically, with a Fleiss agreement score of kappa = 0.642. This Fleiss agreement score is moderate, demonstrating the difficulty of the labeling task. Table 3 shows accuracy and F-scores for the annotators, to evaluate their performance. F-scores take into account both false positives and false negatives, and so can be a more realistic measure than accuracy.

**Table 3:** Annotator accuracy and F-scores.

**Annotator**	**Dataset A**	**Dataset A**	**Dataset B**	**Dataset B**	**Dataset A** + **B**	**Dataset A** + **B**
	**Accuracy**	**F-score**	**Accuracy**	**F-score**	**Accuracy**	**F-score**
1	85.2	83.5	80.0	75.1	83.9	81.4
2	88.2	86.8	87.6	85.8	88.1	86.5
3	78.9	76.4	80.2	79.6	79.2	77.2

*Data after cleaning, 3 categories (promotional, neutral, discouraging)*.

Cohen Kappa scores indicate the agreement between two annotators. The Cohen Kappa scores were 0.640 (Annotator 1-2), 0.586 (Annotator 1-3) and 0.611 (Annotator 2-3). Moderate agreement is seen between each pair of annotators.

### Machine learning model training

The datasets were split: 60% training, 20% validation and 20% testing.

Training data is used to construct a predictive relationship between the data and its label. Validation data is used to build the model, assessing the performance of the model during training. Validation and testing data is used to calculate the accuracy of the model: the model labels the data, then uses the human labels to assess the accuracy of the categorization.

In this blind study, the test data was only used after the model had been selected according to performance on the validation datasets.

A number of Transformer-based machining learning models were chosen and run with the data. The relevant tokenizer was applied in each case.

Models were selected from the open source AI (Artificial Intelligence) library, Hugging Face (33).

Distributed computing was performed using Google CoLab GPU (34).

To get a baseline score for the Transformer-based machine learning models to achieve, a well-established classification model was used, Logistic Regression. Logistic Regression models the probability of an outcome using the statistical method, maximum likelihood estimation. The tokenizer used in this case, to transform text into numbers, was TfidfVectorizer. Term-frequency inverse document frequency (Tfidf), is a measure that is intended to reflect how important a word is to a document. Logistic Regression (35) and TfidfVectorizer (36) achieved an accuracy of 63.22% (F-score of 0.625) (Supplementary Presentation 1).

The Transformer models were trained for a maximum of 13 epochs, where an epoch is a pass of the model over the full training dataset. The performance of the model was evaluated throughout the training procedure using accuracy and loss, where accuracy is the fraction of correct predictions and loss is the mean error of the predictions. If the model's prediction is perfect, the loss is zero; otherwise, the loss is greater than zero. Early stopping was used to monitor accuracy and stop overfitting of the model. Overfitting a model to the training data reduces its ability to generalize and thus predict using other data.

The various Transformer models were tuned with different hyperparameters for best performance on the task datasets. With each hyperparameter combination, the models were run five times, to note the best and range of performance. Through this method, the XLM-RoBERTa-large model was selected for this task.

The XLM-RoBERTa-large model was fine-tuned 5 times with dataset A training data and 5 times with the combined dataset A plus dataset B training data. The best performing model was chosen in each case out of the 5 runs according to F-score from the validation datasets (Supplementary Figure 2). Selecting the model using F-score from the validation data, ensured a blind selection of the model, not biased by results from the test datasets. The accuracy and F-scores are shown for the test datasets.

## Results

The best performing machine learning model was XLM-RoBERTa-large. RoBERTa (A Robustly Optimized BERT Pretraining Approach) is based on BERT. The term XLM indicates it is a cross-lingual (i.e., multi-lingual) model, and the term large indicates the number of encoder layers. The hyperparameters chosen were batch size 16, learning rate 2e-5 and warm-up proportion 0.15. The sequence length was limited to 96 tokens, to ensure that not even the longest tweet in the data was truncated. Token sequence length was checked using XLMRobertaTokenizer (Supplementary Figure 3). The best performing model was trained using the EU-JAV training data and selected according to F-score performance with the EU-JAV validation data. Table 4 shows results for annotators and machine learning models over the full dataset; Table 5 shows results by training dataset and testing dataset.

**Table 4:** Accuracy and F-scores for annotators and ML models over full dataset (dataset A + B).

**Data with 3 categories**	**Accuracy (%)**	**F-score**
**Annotator average**	**83.7**	**0.817**
Annotator 1	83.9	0.814
Annotator 2	88.1	0.865
Annotator 3	79.2	0.772
Logistic regression + TfidfVectorizer	63.2	0.625
Support vector machine (LinearSVC)	64.7	0.645
**Finetuned XLM-RoBERTa-large**	**72.4**	**0.720**

*The bold values indicate annotator average (the baseline result against which ML model results are to be compared) and the XLM-RoBERTa model results*.

**Table 5:** Accuracy and F-scores by training dataset and testing dataset using the fine-tuned XLM-RoBERTa-large model.

**Train dataset**	**Test dataset**	**Accuracy (%)**	**F-score**
Dataset A	Dataset A	72.8	0.713
Dataset A	Dataset B	62.1	0.617
Dataset A	Dataset A+B	70.1	0.689
Dataset A+B	Dataset A	72.8	0.721
Dataset A+B	Dataset B	71.3	0.713
Dataset A+B	Dataset A+B	72.4	0.720

Training on dataset A and testing on dataset A resulted in an accuracy of 72.8% (F-score 0.713). Testing the same model on dataset B, the accuracy dropped to 62.1% (F-score 0.617)—a drop of about 10%.

When the model was retrained with the full dataset (dataset A+B), then the accuracy of the predictions for dataset A remained at 72.8% (F-score increased slightly to 0.721) and the accuracy for dataset B increased to 71.3% (F-score 0.713).

The dataset B was too small to be used for independent training. However, combining the two datasets dramatically improved the model performance for dataset B, bringing the accuracy for the smaller more recent dataset into line with that over the larger original dataset.

This indicates that model retraining with not just more data, but also more recent data, proves advantageous and helps the model keep up-to-date with the natural change in task-specific language.

The accuracy of the model trained and tested on the combined datasets was 72.4% (F-score = 0.720).

Confusion matrices aid evaluation of the classification accuracy. The diagonal in a confusion matrix shows how well the predicted label matches the human annotator label, i.e., where the tweet has been “correctly” identified by the model. The off-diagonal elements are those that are mislabeled by the model. Normalization results in each row of the matrix adding up to 1, thus percentages of correctly or wrongly identified elements are shown.

**Figure 2:**
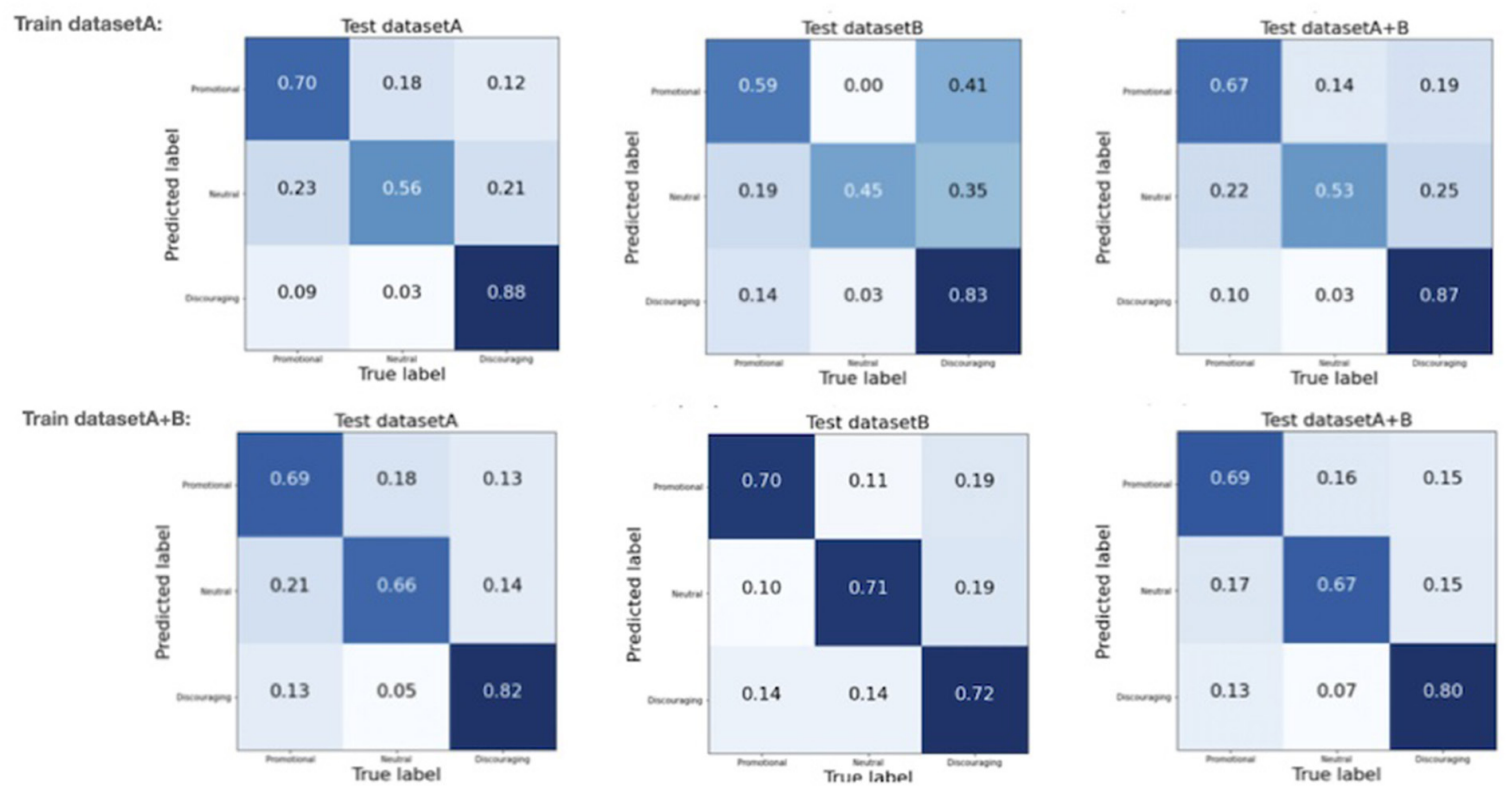
Normalized confusion matrices for the final selected fine-tuned model. Top row: XLM-Roberta-large model trained on dataset A. Bottom row: XLM-Roberta-large model trained on the combined datasets (dataset A+B). On the left, the matrices when the model is tested on dataset A. In the centre, the matrices when the model is tested on dataset B. On the right, the matrices when the model is tested on the combined datasets.

Figure 2 shows normalized confusion matrices. The top row is for the XLM-Roberta-large model trained on dataset A, the bottom row for the XLM-Roberta-large model trained on the combined datasets (dataset A+B). On the left, the matrices when the model is tested on dataset A, in the center, the matrices when the model is tested on dataset B, and on the right, the matrices when the model is tested on the combined datasets. The confusion matrices show that discouraging tweets are the easiest out of the three categories to identify. Training on the combined datasets helps the model differentiate between the three categories in dataset A, even if it does not increase the overall accuracy of the model: the confusion matrices when testing on dataset A show a marked increase in correctly identifying neutral tweets (56–66%) with a slight decrease in correctly identifying promotional and discouraging tweets (70–69% and 88–82%, respectively). The confusion matrices when testing on dataset B show the benefits of retraining on more recent data: an increase from 59 to 70% for correctly identifying promotional tweets, and an increase from 45 to 71% for correctly identifying neutral tweets. A slight reduction in performance for correctly identifying discouraging tweets is noted (from 83 to 72%), balanced by an increase in mislabeled neutral tweets (0.03–0.14%). These results support the suggestion that model retraining with more recent data helps the model in the classification task, which is likely not just due to the increased dataset size, but also familiarity with more recent task-specific language.

## Discussion

We present a NLP machine learning Transformer-based model aimed at automatically classifying the stance of Italian vaccine-related tweets with a level of accuracy of 72.4% and with an F-score of 0.720. We show a reduction of the model's accuracy when tested on more recent data compared to that used for training the model, and an improvement when including more recent data in the training.

Dataset A was collected between November 2019 and June 2020. Dataset B was collected between April 2021 and September 2021. The annotators noticed that the language and focus within the social discourse about vaccination evolved over the time of study—new words entered the conversation, such as COVID-19 vaccine brand names or words referring to political initiatives, e.g., “green pass”.

We saw a decrease of the accuracy and of the F-score for the model trained on the 2019–2020 data, when we tested it on the 2021 data. The performance of the model increased when testing on the 2021 data (both for accuracy and F-score) when we included 2021 data in the training set.

The selected machine learning Transformer-based model, XLM-RoBERTa-large (37), was published in November 2019. It was pre-trained on 2.5TB of filtered CommonCrawl data (38) containing 100 languages. However, SARS-CoV-2 was first confirmed to have spread to Italy on January 31, 2020, thus the model had not seen any COVID-19 related conversations or COVID-19 specific language. Pre-training the XLM-RoBERTa-large model on unlabeled 2020 Twitter data may well increase its performance on this task.

Different tokenizers have different vocabularies. The model we selected for this task, XLM-RoBERTa-large, uses a tokenizer with a vocabulary size of 250,002. For comparison, another popular multilingual model [bert-base-multilingual-uncased (39)] uses a vocabulary size of 105,879, just under half that of the XLM-RoBERTa-large. A mono-lingual model pre-trained specifically for the task of the COVID-19 discussion on Twitter, covid-twitter-bert-v2 (40), uses vocabulary from English BERT with a size of 30,522. Words such as “mask,” “confinement,” and “vaccine” are in its vocabulary whereas they are not present in the XLM-RoBERTa vocabulary. Thus, a limitation of the XLM-RoBERTa tokenizer, despite its size, is that it was released in 2019 and so COVID-19 specific words and their COVID-19 context-specific meanings are not in its vocabulary. However, words such as “isolation” and “virus” are in its vocabulary and perhaps explain its reasonable performance on this task.

Manual stance coding is very time consuming and is subjective in nature. One of the strengths of this study was the precision of the data labeling phase, which was carried out by a multidisciplinary group including physicians with experience in vaccines and social scientists with experience in internet studies. The labeling process followed a structured protocol, training sessions were carried out before the labeling started and weekly discussions were held to fine-tune the classification process among the researchers involved. We decided to focus on data quality rather than quantity. Thus, a corpus size of 1,736 unique tweets, carefully labeled, was considered sufficient to make a meaningful study. Subjectivity was addressed by using three independent coders and discussing discrepancies.

As previously discussed, sentiment analysis is not sufficiently nuanced to have a meaningful description of vaccine confidence on social media data (see Supplementary Figure 4). Other studies have addressed vaccine stance classification in Italian, as well as in other languages. D'Andrea et al. (41) trained a model for vaccine stance classification on Twitter with reference to the vaccination topic in Italy. To this aim, the research group used a set of 693 tweets published between September 2016 and January 2017. Accuracy, achieved by classical ML methods, namely Bag Of Words text representation (vectorization) and a Support Vector Machine (SVM) classifier, was 64.84%. The researchers pointed out the risk of deterioration of the classification models, given the potential changes of the vaccine conversation over time, possibly due to the occurrence of specific social context-related events.

In order to understand how a model similar to the one used by D'Andrea et al. would perform on our data, we used a SVM model to classify the EU-JAV data and achieved 63.51% accuracy. Classical ML models achieved lower accuracy than the Transformer models on the EU-JAV data, confirming the recent improvements in NLP using Transformer models. BERT was created and published by Google in 2018 and is now ubiquitous in NLP (26).

Kummervold et al. (29) studied the stance of tweets on maternal vaccination using Transformer-based machine learning models and showed that machine learning models can achieve similar accuracy to a single annotator. Their best model achieved an accuracy of 81.8% (F-score = 0.776) compared to the agreed score between three annotators, and the accuracies of the individual annotators compared to the final score were 83.3, 77.9, and 77.5%. Their data was collected over 6 months, between November 2018 and April 2019, and, after cleaning, consisted of 2,722 tweets. In our study, data after cleaning consisted of 1,736 tweets (64% of the size of the Kummervold dataset), collected over a total time period of 22 months. Our annotators demonstrated very similar accuracy, which seems reasonable considering the similarity in task (79.2, 83.9, and 88.1%). However, our fine tuned model did not achieve the same level of accuracy as Kummervold's, 72.4% (F-score = 0.720) cf 81.8% (F-score = 0.776). The lower accuracy of our model might be in part due to the reduced dataset size, or to the drift in stance over the extended time period of data collection. The Kummervold study was carried out in English (non-English tweets were translated into English using a Google Translate script), whereas our study was carried out in Italian. NLP models have seen more work in English (42). This is highlighted by the models available on Hugging Face, an open source library of NLP models, where there are around 15,000 mono-lingual models in English and just 10,000 mono-lingual models in all other languages. This may be due in part to the availability of data and financial resources. Other language models and multilingual models are seeing advances but still seem to be behind in development and performance.

Another possible explanation of the difference in the accuracies between Kummervold's and our model could be that Twitter in Italy is used mainly by professional people, e.g., experts, journalists and public institutions, participating in public conversations (43). Most of the conversations are intellectual and often both language and content are quite sophisticated and rhetorical. This could make both the annotators' and the model's classification task harder than that for other languages. This language complexity is the reason why it was decided to keep the tweets in Italian and not to translate them into English.

Kumar et al. (44) used COVID-Twitter-BERT, a model pretrained on COVID Twitter data, achieving an F1-score of 0.532 and an accuracy of 0.532, demonstrating that stance classification is not straightforward. The dataset contained 1,010 tweets of neutral, 991 tweets of ProVax, and 791 tweets of AntiVax classes. The COVID-Twitter-BERT model is in English and so not appropriate for our study.

Cotfas et al. (45) studied a dataset of 3,249 tweets selected from 5,030,866 tweets in English collected from Twitter between December 8, 2020 and January 7, 2021. The best performing classical machine learning classifier was a Support Vector Machine with accuracy 72.19%. The best performing deep learning classifier was RoBERTa with accuracy 78.63%. The accuracy in this study is slightly better than we achieve, as would be expected from a larger dataset (about twice the size). Again, the model used was pre-trained for the English language.

Lemmens et al. (46) describes development of CoNTACT, a Dutch language model adapted to the domain of COVID-19 tweets. The model was pre-trained on 2.8 M dutch tweets related to COVID-19. CoNTACT achieved an F1-score of 77.1 improving on its base model RobBERT, a Dutch RoBERTa model (F1-score 75.1). Models that are pre-trained on a specific target domain show improved performance. This could be considered for future Italian activities to develop better models focused on Italian text.

An important issue that emerges from our results is the need for retraining of the model through time. This has been already suggested by other authors, but our results clearly support this recommendation. The COVID-19 vaccine roll-out disrupted the characteristics of the language and content of the social media conversation on vaccines, and this is reflected in the performance of our model when tested on newer data. A continuous process of model re-training is needed to keep up with the evolving characteristics of the vaccine discourse.

The use of non-conventional data streams can have a crucial impact on public health activities. Google search data, combined with historical surveillance data and data from electronic health records, have been used to forecast influenza epidemic trends (47), and prediction models based on Google search data have been developed for dengue (48) and Ebola (49). Information spontaneously provided by users on social media could be used to complement pharmacovigilance activities (50) and syndromic surveillance activities (51). Twitter reactions have been found to be correlated with COVID-19 epidemic waves in Japan (52).

As pointed out by Aiello et al. (17) in an analysis of the way digital data could improve public health, “improving population health depends on effective communication and interventions”. Constant monitoring of vaccine stance on social media through NLP can enable public health agencies to have a real-time understanding of the favorability toward vaccines among social media users. We acknowledge that social media users are not a representative sample of the whole population. Though, as the use of social media is constantly rising, and this rise has been even more consistent during the COVID-19 pandemic, social media users represent a wide part of the population and a potential target for health communication initiatives. Moreover, the importance of social media is not limited to its users, as stories and news circulating on social media affect the way health-related themes are discussed also on mainstream media (53, 54), which still remains the most used information source (55), especially for age groups typically not familiar with social media channels.

Through vaccine stance analysis, popular narratives and misinformation/disinformation circulating in discouraging posts could be quickly identified, thus triggering actions by institutions to counteract the dangers posed by fake news. Moreover, automatic vaccine-stance analysis could be used for monitoring the effect of important events (e.g., rollout of a new vaccine) or the impact of vaccine promotion campaigns on vaccine acceptance in almost real time. Finally, real-time data on vaccine stance could be combined with traditional data on vaccine acceptance (e.g., obtained through national surveys) and with vaccine coverage figures to better understand the relationship between opinions expressed online and the actual behavior of the population.

NLP is constantly evolving. GPT-3 (Generative Pre-trained Transformer 3) (56) can perform reading comprehension and writing tasks at a near-human level. Such powerful models with critical thinking and logic skills promise applications in many areas (57) including translation and multilingualism (58). Work is on-going regards sarcasm detection (59–61) and understanding complex opinions (62) (e.g., positive stance toward vaccines but negative stance toward institutions or policies) with the aim of more nuanced interpretation of people's opinion. Future models will focus on zero-shot, one-shot and few-shot learning, where models will classify objects from zero, one, or very few, samples (63, 64).

Our study has a number of limitations. First, our data was limited by what could be exported from Twitter. The number of tweets per week or per month is not constant (shown in Supplementary Figure 5) and we have no control over the selection of data over the time period. Moreover, Twitter users in Italy only represent a small percentage of the population (5–13%) (43), and have their own language characteristics, therefore the applicability of the model to other kinds of text should be investigated before being used. Three annotators labeled the data. However, one of the annotators was leading the analysis, so when there was disagreement and hence discussion of how to label a tweet, their point of view may have been weighted unconsciously. The annotator agreements with the final label indicate a slight bias toward the lead annotator. In any group this weighting is likely to occur and would be difficult to remove. The tweets that the model labeled differently to the annotators were studied. In some instances, after further consideration, the model label was thought more correct than the annotator label. In future research, one could consider removing such “difficult to label” tweets from the dataset or use the model as a fourth annotator.

## Conclusion

Our results on selecting and fine tuning a NLP machine learning model to classify vaccine-related tweets according to their stance show that retraining a model on recent social media discourse data improves the model's performance on that data. This is likely not just due to the increased dataset size, but also to familiarity with the evolved terminology and public stance. It is advisable therefore to periodically fine-tune NLP social media monitoring models to keep in step with the natural change of language within a subject over time.

Understanding public stance toward vaccines is crucial for governments and public health agencies to help guide them in the development of educational campaigns and targeted communication. Machine learning enabled social media analysis should be considered alongside conventional methods of assessing public attitude, for its potential to obtain a close to real-time assessment of public confidence. Machine learning based vaccine stance monitoring could help institutions to address the concerns of vaccine skeptics, to develop more effective policies and communication strategies, and to monitor their impact, with the aim of maximizing trust in and uptake of vaccines. Future research should focus on studying the performance of the most recent NLP models, which will allow for a more accurate and nuanced description of vaccine stance on social media.

## Data Availability Statement

The datasets presented in this study can be found in online repositories. The names of the repository/repositories and accession number(s) can be found below: https://github.com/FrGes/EU-JAV/blob/main/datasetA_test_3categories.csv (Dataset A test data); https://github.com/FrGes/EU-JAV/blob/main/datasetB_test_3categories.csv (Dataset B test data); https://huggingface.co/FrGes/xlm-roberta-large-finetuned-EUJAV-datasetA (Machine learning model finetuned on Dataset A); https://huggingface.co/FrGes/xlm-roberta-large-finetuned-EUJAV-datasetAB (Machine learning model finetuned on Dataset A+B).

## Author contributions

SC, PEK, and FG conceived the study. SC conducted the analysis (data cleaning, model selection, model fine tuning, and evaluating the results) and drafted the manuscript. PEK supervised the machine learning activities. FG coordinated the study and drafted the manuscript. LP, FC, and BL contributed to the tweet classification. IC headed up data management. PEK, AF, MR, CR, and AT revised the manuscript. All authors contributed to the article and approved the submitted version.

## Funding

The study was funded by Grant No. 801495 of the Consumer, Health, Agriculture and Food Executive Agency (CHAFEA). PEK was funded by the European Commission for the call H2020-MSCA-IF-2017 and the funding scheme MSCA-IF-EF-ST for the Vaccine Media Analytics project (Grant Agreement ID: 797876).

## Conflict of interest

AT received grants from MSD for invited lectures. The remaining authors declare that the research was conducted in the absence of any commercial or financial relationships that could be construed as a potential conflict of interest.

## Publisher's note

All claims expressed in this article are solely those of the authors and do not necessarily represent those of their affiliated organizations, or those of the publisher, the editors and the reviewers. Any product that may be evaluated in this article, or claim that may be made by its manufacturer, is not guaranteed or endorsed by the publisher.

## Abbreviations

NLP: Natural Language Processing
BERT: Bidirectional Encoder Representations from Transformers
EU-JAV: European Joint Action on Vaccination
Tfidf: Term-frequency inverse document frequency
RoBERTa: A Robustly Optimized BERT Pretraining Approach
SVM: Support Vector Machine.

## References

[1] MacDonald NE SAGE SAGE Working Group on Vaccine Hesitancy. Vaccine hesitancy: definition, scope and determinants. Vaccine. (2015) 33:4161–4. 10.1016/j.vaccine.2015.04.036 25896383

[2] Ten Health Issues WHO Will Tackle This Year. https://www.who.int/news-room/spotlight/ten-threats-to-global-health-in-2019 (accessed March 11, 2022).

[3] Omer SB Salmon DA Orenstein WA deHart MP Halsey N. Vaccine refusal, mandatory immunization, and the risks of vaccine-preventable diseases. N Engl J Med. (2009) 360:1981–8. 10.1056/NEJMsa0806477 19420367

[4] Phadke VK Bednarczyk RA Salmon DA Omer SB. Association between vaccine refusal and vaccine-preventable diseases in the united states: a review of measles and pertussis. JAMA. (2016) 315:1149–58. 10.1001/jama.2016.1353 26978210 PMC5007135

[5] Solís Arce JS Warren SS Meriggi NF Scacco A McMurry N Voors M. COVID-19 vaccine acceptance and hesitancy in low- and middle-income countries. Nat Med. (2021) 27:1385–94. 10.1038/s41591-021-01454-y 34272499 PMC8363502

[6] Tenforde MW Self WH Adams K Gaglani M Ginde AA McNeal T. Association between mRNA vaccination and COVID-19 hospitalization and disease severity. JAMA. (2021) 326:2043. 10.1001/jama.2021.19499 34734975 PMC8569602

[7] Tenforde MW Self WH Gaglani M Ginde AA Douin DJ Talbot HK. Effectiveness of mRNA vaccination in preventing COVID-19–associated invasive mechanical ventilation and death — United States, March 2021–January 2022. MMWR Morb Mortal Wkly Rep. (2022) 71:459–65. 10.15585/mmwr.mm7112e1 35324878 PMC8956334

[8] Olivera Mesa D Hogan AB Watson OJ Charles GD Hauck K Ghani AC. Modelling the impact of vaccine hesitancy in prolonging the need for non-pharmaceutical interventions to control the COVID-19 pandemic. Commun Med. (2022) 2:14. 10.1038/s43856-022-00075-x 35603311 PMC9053271

[9] Nsoesie EO Cesare N Müller M Ozonoff A. COVID-19 misinformation spread in eight countries: exponential growth modeling study. J Med Internet Res. (2020) 22:e24425. 10.2196/24425 33264102 PMC7744144

[10] Scharrer L Rupieper Y Stadtler M Bromme R. When science becomes too easy: science popularization inclines laypeople to underrate their dependence on experts. Public Underst Sci. (2017) 26:1003–18. 10.1177/0963662516680311 27899471

[11] Ford AJ Alwan NA. Use of social networking sites and women's decision to receive vaccinations during pregnancy: a cross-sectional study in the UK. Vaccine. (2018) 36:5294–303. 10.1016/j.vaccine.2018.07.022 30055969

[12] Mohanty S Leader AE Gibeau E Johnson C. Using Facebook to reach adolescents for human papillomavirus (HPV) vaccination. Vaccine. (2018) 36:5955–61. 10.1016/j.vaccine.2018.08.060 30172634

[13] Hou Z Tong Y Du F Lu L Zhao S Yu K. Assessing COVID-19 vaccine hesitancy, confidence, and public engagement: a global social listening study. J Med Internet Res. (2021) 23:e27632. 10.2196/27632 34061757 PMC8202656

[14] Islam MS Kamal A-HM Kabir A Southern DL Khan SH Hasan SMM. COVID-19 vaccine rumors and conspiracy theories: The need for cognitive inoculation against misinformation to improve vaccine adherence. PLoS ONE. (2021) 16:e0251605. 10.1371/journal.pone.0251605 33979412 PMC8115834

[15] European Centre for Disease Prevention Control. Systematic Scoping Review on Social Media Monitoring Methods and Interventions Relating to Vaccine Hesitancy. LU: Publications Office (2019). Available online at: https://data.europa.eu/doi/10.2900/260624 (accessed October 27, 2021).

[16] Karafillakis E Martin S Simas C Olsson K Takacs J Dada S. Methods for social media monitoring related to vaccination: systematic scoping review. JMIR Public Health Surveill. (2021) 7:e17149. 10.2196/17149 33555267 PMC7899807

[17] Aiello AE Renson A Zivich PN. Social media- and internet-based disease surveillance for public health. Annu Rev Public Health. (2020) 41:101–18. 10.1146/annurev-publhealth-040119-094402 31905322 PMC7959655

[18] Managing the COVID-19 Infodemic: Promoting Healthy Behaviours and Mitigating the Harm From Misinformation and Disinformation. Available online at: https://www.who.int/news/item/23-09-2020-managing-the-covid-19-infodemic-promoting-healthy-behaviours-and-mitigating-the-harm-from-misinformation-and-disinformation (accessed July 4, 2022).

[19] Tangcharoensathien V Calleja N Nguyen T Purnat T D'Agostino M Garcia-Saiso S. Framework for managing the COVID-19 infodemic: methods and results of an online, crowdsourced WHO technical consultation. J Med Internet Res. (2020) 22:e19659. 10.2196/19659 32558655 PMC7332158

[20] Louis A. Natural language processing for social media. Comput Linguist. (2016) 42:833–36. 10.1162/COLI_r_00270

[21] Mikolov T Chen K Corrado G Dean J. Efficient estimation of word representations in vector space. arXiv [Preprint]. (2013). arXiv:1301.3781. 10.48550/arXiv.1301.3781

[22] Mikolov T Sutskever I Chen K Corrado GS Dean J. Distributed representations of words and phrases and their compositionality. Adv Neural Inf Process Syst. (2013) 26:3111–119.31840584

[23] Pennington J Socher R Manning CD. Glove: global vectors for word representation. In: Proceedings of the 2014 Conference on Empirical Methods in Natural Language Processing (EMNLP). (2014). p. 1532–43.

[24] Kleene SC. Representation of events in nerve nets and finite automata. Autom Stud. (1956) 34:3–41. 10.1515/9781400882618-002

[25] Bengio Y. Learning deep architectures for AI. Found Trends Mach Learn. (2009) 2:1–127. 10.1561/2200000006

[26] Devlin J Chang M-W Lee K Toutanova K. BERT: Pre-training of Deep Bidirectional Transformers for Language Understanding. ArXiv181004805 Cs. (2019). Available online at: http://arxiv.org/abs/1810.04805 (accessed April 20, 2022).

[27] Mohammad SM Sobhani P Kiritchenko S. Stance and sentiment in tweets. ACM Trans Internet Technol. (2017) 17:1–23. 10.1145/3003433

[28] Martin S Kilich E Dada S Kummervold PE Denny C Paterson P Larson HJ. “Vaccines for pregnant women…?! Absurd” – Mapping maternal vaccination discourse and stance on social media over six months. Vaccine. (2020) 38:6627–37. 10.1016/j.vaccine.2020.07.072 32788136

[29] Kummervold PE Martin S Dada S Kilich E Denny C Paterson P. Categorizing vaccine confidence with a transformer-based machine learning model: analysis of nuances of vaccine sentiment in Twitter discourse. JMIR Med Inform. (2021) 9:e29584. 10.2196/29584 34623312 PMC8538052

[30] The Pandemic Is Changing the English Language - CNN. Available online at: https://edition.cnn.com/2020/10/16/health/english-language-changing-coronavirus-wellness-partner/index.html (accessed April 20, 2022).

[31] EU-JAV - European Joint Action on Vaccination. EU-JAV. Available online at: https://eu-jav.com/ (accessed October 27, 2021).

[32] Kim Y Huang J Emery S. Garbage in, garbage out: data collection, quality assessment and reporting standards for social media data use in health research, infodemiology and digital disease detection. J Med Internet Res. (2016) 18:e41. 10.2196/jmir.4738 26920122 PMC4788740

[33] Hugging Face – The AI Community Building the Future. Available online at: https://huggingface.co/ (accessed April 20, 2022).

[34] Google Colab. Available online at: https://research.google.com/colaboratory/faq.html (accessed April 20, 2022).

[35] sklearn.linear_model.LogisticRegression. Scikit-Learn. Available online at: https://scikit-learn.org/stable/modules/generated/sklearn.linear_model.LogisticRegression.html (accessed April 20, 2022).

[36] sklearn.feature_extraction.text.TfidfVectorizer. Scikit-Learn. Available online at: https://scikit-learn.org/stable/modules/generated/sklearn.feature_extraction.text.TfidfVectorizer.html (accessed April 20, 2022).

[37] xlm-roberta-large. Hugging Face. Available online at: https://huggingface.co/xlm-roberta-large (accessed April 20, 2022).

[38] *Common Crawl*. Available online at: https://commoncrawl.org/ (accessed April 22, 2022).

[39] bert-base-multilingual-uncased · *Hugging Face*. Available online at: https://huggingface.co/bert-base-multilingual-uncased (accessed April 20, 2022).

[40] digitalepidemiologylab/covid-twitter-bert-v2 · *Hugging Face*. Available online at: https://huggingface.co/digitalepidemiologylab/covid-twitter-bert-v2 (accessed April 20, 2022).

[41] D'Andrea E Ducange P Bechini A Renda A Marcelloni F. Monitoring the public opinion about the vaccination topic from tweets analysis. Expert Syst Appl. (2019) 116:209–26. 10.1016/j.eswa.2018.09.00926013683

[42] Tracking, Progress in Natural Language Processing. NLP-Prog. Available online at: http://nlpprogress.com/ (accessed April 20, 2022).

[43] Alessandra R Gentile MM Bianco DM. Who tweets in Italian? Demographic characteristics of Twitter users. In: Petrucci A, Racioppi F, Verde R, editors. New Statistical Developments in Data Science. Springer Proceedings in Mathematics & Statistics. Cham: Springer International Publishing (2019). p. 329–44. 10.1007/978-3-030-21158-5_25

[44] Kumar A Roy PK Singh JP. Bidirectional Encoder Representations from Transformers for the COVID-19 Vaccine Stance Classification (2021). Available online at: http://ceur-ws.org/Vol-3159/T8-4.pdf

[45] Cotfas L-A Delcea C Gherai R. COVID-19 vaccine hesitancy in the month following the start of the vaccination process. Int J Environ Res Public Health. (2021) 18:10438. 10.3390/ijerph181910438 34639738 PMC8508534

[46] Lemmens J Van Nooten J Kreutz T Daelemans W. CoNTACT: A Dutch COVID-19 adapted BERT for vaccine hesitancy and argumentation detection. arXiv [Preprint]. (2022). arXiv: 2203.07362. 10.48550/arXiv.2203.07362

[47] Yang S Santillana M Brownstein JS Gray J Richardson S Kou SC. Using electronic health records and Internet search information for accurate influenza forecasting. BMC Infect Dis. (2017) 17:332. 10.1186/s12879-017-2424-7 28482810 PMC5423019

[48] Althouse BM Ng YY Cummings DAT. Prediction of dengue incidence using search query surveillance. PLoS Negl Trop Dis. (2011) 5:e1258. 10.1371/journal.pntd.0001258 21829744 PMC3149016

[49] Alicino C Bragazzi NL Faccio V Amicizia D Panatto D Gasparini R. Assessing Ebola-related web search behaviour: insights and implications from an analytical study of Google Trends-based query volumes. Infect Dis Poverty. (2015) 4:54. 10.1186/s40249-015-0090-9 26654247 PMC4674955

[50] Lardon J Bellet F Aboukhamis R Asfari H Souvignet J Jaulent M-C. Evaluating Twitter as a complementary data source for pharmacovigilance. Expert Opin Drug Saf. (2018) 17:763–74. 10.1080/14740338.2018.1499724 29991282

[51] Yousefinaghani S Dara R Poljak Z Bernardo TM Sharif S. The assessment of Twitter's potential for outbreak detection: avian influenza case study. Sci Rep. (2019) 9:18147. 10.1038/s41598-019-54388-4 31796768 PMC6890696

[52] Tran V Matsui T. Tweet analysis for enhancement of COVID-19 epidemic simulation: a case study in Japan. Front Public Health. (2022) 10:806813. 10.3389/fpubh.2022.806813 35433607 PMC9008370

[53] Jang SM Mckeever BW Mckeever R Kim JK. From social media to mainstream news: the information flow of the vaccine-autism controversy in the US, Canada, and the UK. Health Commun. (2019) 34:110–7. 10.1080/10410236.2017.1384433 29028371

[54] Skogerbø E Bruns A Quodling A Ingebretsen T. Social media and sourcing in mainstream journalism. Routledge Companion Soc Media Polit. (2015) 104:104–20. 10.4324/9781315716299-831469337

[55] Ali SH Foreman J Tozan Y Capasso A Jones AM DiClemente RJ. Trends and predictors of COVID-19 information sources and their relationship with knowledge and beliefs related to the pandemic: nationwide cross-sectional study. JMIR Public Health Surveill. (2020) 6:e21071. 10.2196/21071 32936775 PMC7546863

[56] *GPT-3: Language Models are Few-Shot Learners*. OpenAI (2022). Available online at: https://github.com/openai/gpt-3 (accessed July 8, 2022).

[57] Korngiebel DM Mooney SD. Considering the possibilities and pitfalls of generative pre-trained transformer 3 (GPT-3) in healthcare delivery. NPJ Digit Med. (2021) 4:93. 10.1038/s41746-021-00464-x 34083689 PMC8175735

[58] GPT-3 Powers the Next Generation of Apps. OpenAI. (2021). Available online at: https://openai.com/blog/gpt-3-apps/ (accessed July 8, 2022).

[59] mrm8488/t5-base-finetuned-sarcasm-twitter. Hugging Face. Available online at: https://huggingface.co/mrm8488/t5-base-finetuned-sarcasm-twitter (accessed July 7, 2022).

[60] Oprea S Magdy W. iSarcasm: A dataset of intended sarcasm. arXiv [Preprint]. (2019). arXiv:1911.03123. 10.48550/ARXIV.1911.03123

[61] Ashwitha A Shruthi G Shruthi HR Upadhyaya M Ray AP Manjunath TC. Sarcasm detection in natural language processing. Mater Today Proc. (2021) 37:3324–31. 10.1016/j.matpr.2020.09.124

[62] Liu E Cui C Zheng K Neubig G. Testing the ability of language models to interpret figurative language. arXiv [Preprint]. (2022). arXiv:2204.12632. 10.48550/ARXIV.2204.12632

[63] Chang M-W Ratinov L-A Roth D Srikumar V. Importance of semantic representation: dataless classification. In: Aaai. (2008). p. 830–5.

[64] Sanh V Webson A Raffel C Bach SH Sutawika L Alyafeai Z. Multitask prompted training enables zero-shot task generalization. arXiv [Preprint]. (2021). arXiv:2110.08207. 10.48550/ARXIV.2110.08207

