# Supplementary material for: Understanding the vaccine stance of Italian tweets and addressing language changes through the COVID-19 pandemic: Development and validation of a machine learning model
Source: Front Public Health. 2022 Jul 29;10:948880. doi: 10.3389/fpubh.2022.948880 (PMC9372360; doi:10.3389/fpubh.2022.948880)

Baseline

Logistic Regression was chosen as the benchmark since it is fast and efficient to train and run. Similar performance was seen with other traditional classification models.

**Model**	**Accuracy (%)**	**F-score**
Logistic Regression	0.6321	0.6250
Support Vector Machine (LinearSVC)	0.6351	0.6321
Gaussian Process Classifier ((1.0 * RBF(1.0))	0.6494	0.6374

Confusion Matrix for Logistic Regression trained and tested with dataset A+B

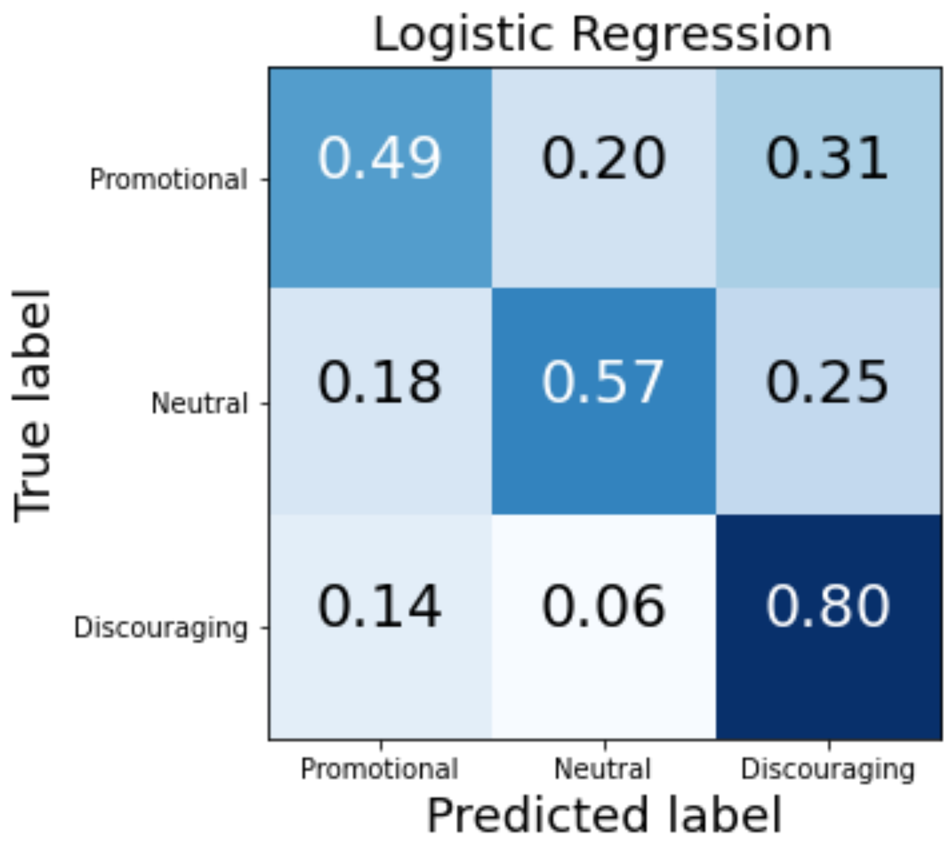

Supplement: Supplementary file 1 [file Presentation_1.PDF]
