# Supplementary material for: Understanding the vaccine stance of Italian tweets and addressing language changes through the COVID-19 pandemic: Development and validation of a machine learning model
Source: Front Public Health. 2022 Jul 29;10:948880. doi: 10.3389/fpubh.2022.948880 (PMC9372360; doi:10.3389/fpubh.2022.948880)
Supplement: Supplementary file 2 [file Image_1.PDF]

Labelling the data. 5 categories reduced to 3.

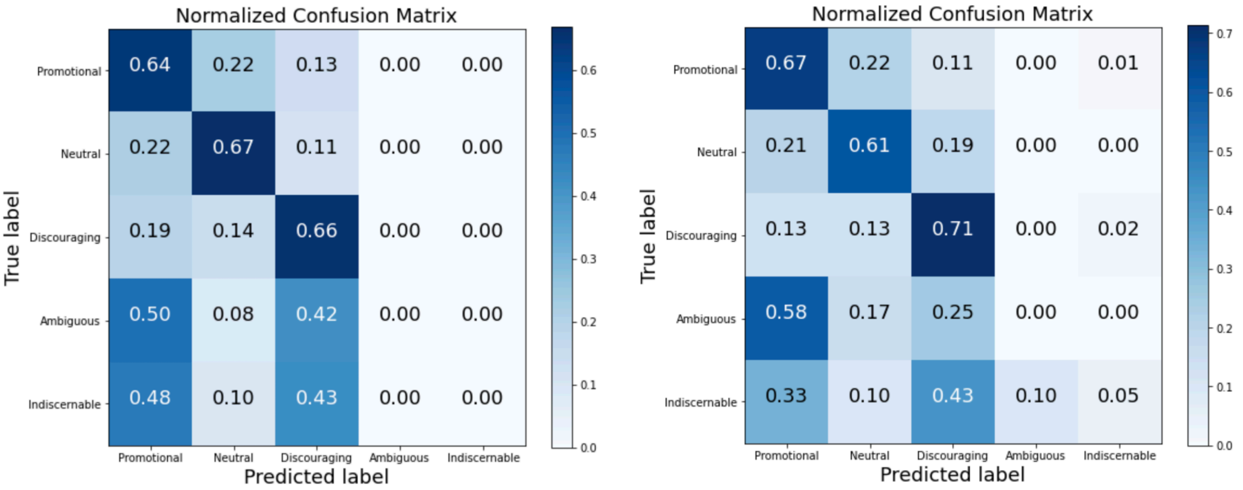

Confusion matrices for 5 stance categories.

When the model was allowed 5 stance categories (promotional, neutral, discouraging, ambiguous, indiscernible), it did not predict many (if any) data points to be ambiguous or indiscernible. This could be because these two categories of data contributed only 9% of the total data (5% dataset A and 17% dataset B), and so insufficient to be meaningful. Or perhaps the tweets were so inconsistent in themselves that it was difficult for the model to see a pattern to group them. This motivated the decision to just include three categories of stance in the study.
