# Supplementary material for: Understanding the vaccine stance of Italian tweets and addressing language changes through the COVID-19 pandemic: Development and validation of a machine learning model
Source: Front Public Health. 2022 Jul 29;10:948880. doi: 10.3389/fpubh.2022.948880 (PMC9372360; doi:10.3389/fpubh.2022.948880)
Supplement: Supplementary file 3 [file Image_2.PDF]

Model selection

The best performing model was chosen in each case out of the 5 runs according to F-score from the validation datasets, since there fluctuation was seen in the accuracy.

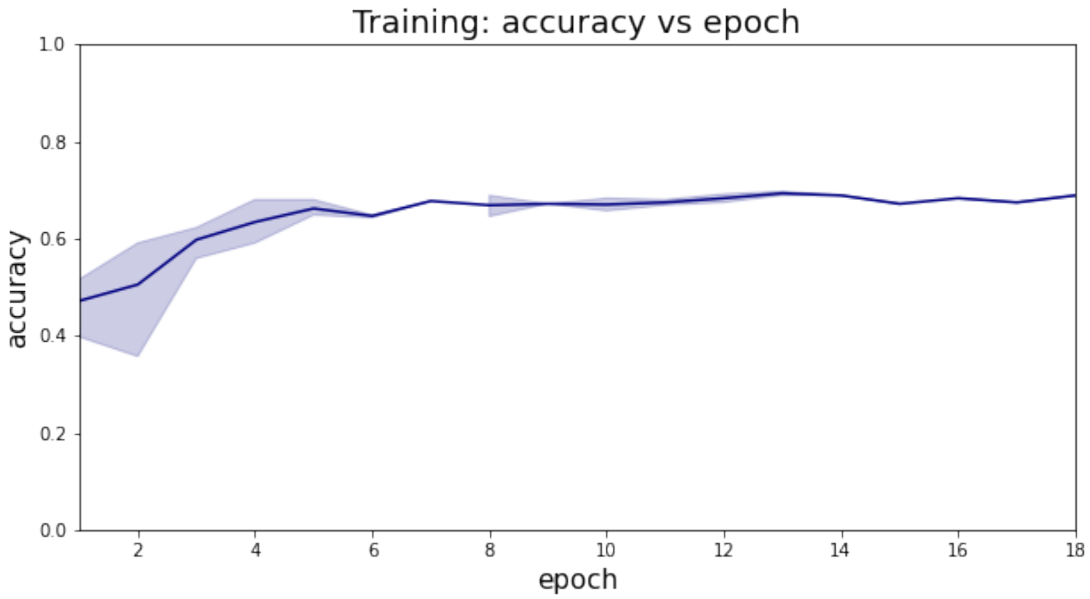

Training accuracy is shown at the end of each epoch for 3 fine-tuning runs of the XLM-RoBERTa-large model, with no early stopping. The accuracy varies between each run, with the range of accuracy decreasing as the epochs increase. The average accuracy is shown by the blue solid line and range of values shown by the shaded region. This accuracy variation was one motivating factor in running the model 5 times using validation data to make the final model selection.
