# Supplementary material for: Understanding the vaccine stance of Italian tweets and addressing language changes through the COVID-19 pandemic: Development and validation of a machine learning model
Source: Front Public Health. 2022 Jul 29;10:948880. doi: 10.3389/fpubh.2022.948880 (PMC9372360; doi:10.3389/fpubh.2022.948880)
Supplement: Supplementary file 4 [file Image_3.PDF]

Data processing

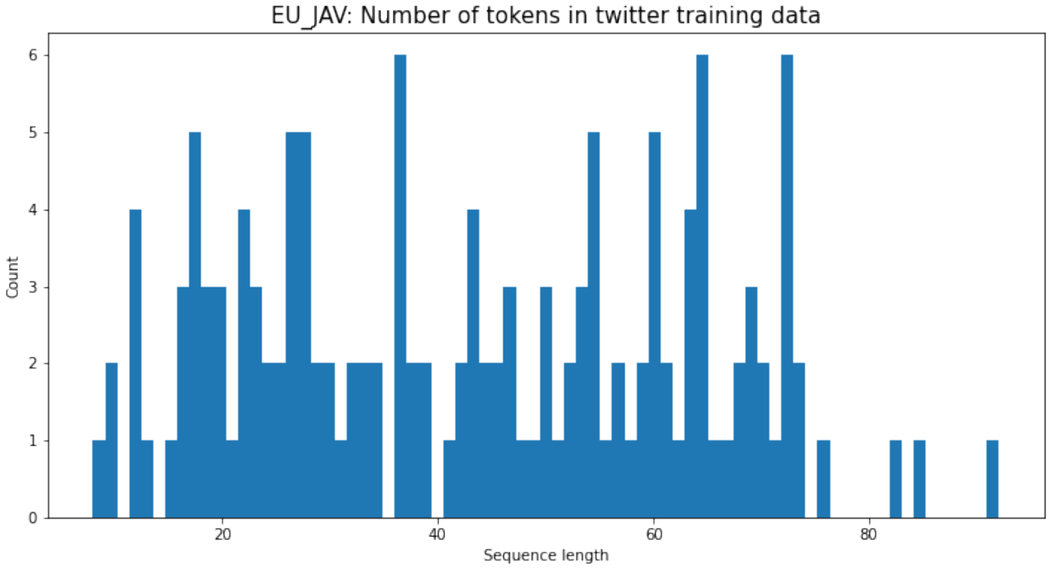

Number of tokens in the training dataset. The tweets were tokenized using the model specific tokenizer. The figure shows the number of tokens in the training dataset when using XLMRobertaTokenizer.
