# Supplementary material for: Understanding the vaccine stance of Italian tweets and addressing language changes through the COVID-19 pandemic: Development and validation of a machine learning model
Source: Front Public Health. 2022 Jul 29;10:948880. doi: 10.3389/fpubh.2022.948880 (PMC9372360; doi:10.3389/fpubh.2022.948880)
Supplement: Supplementary file 5 [file Image_4.PDF]

Sentiment vs Stance

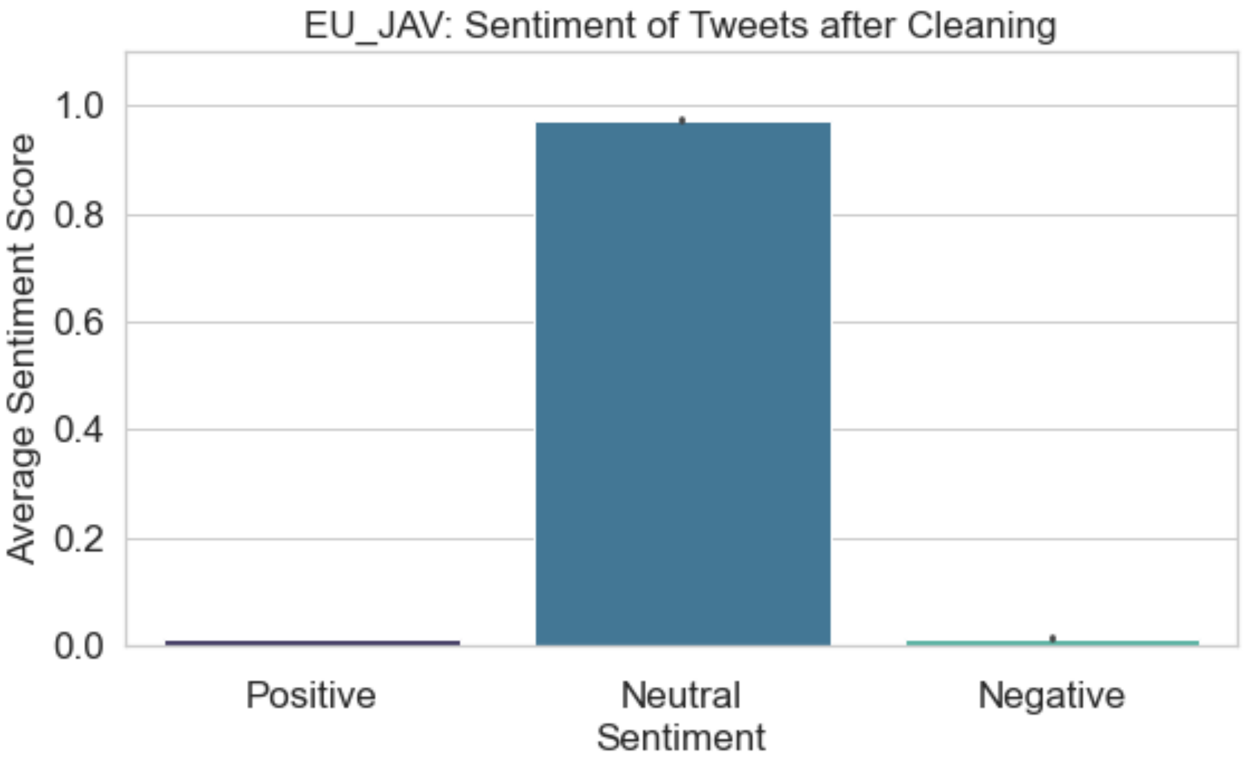

Using Python Natural Language Toolkit SentimentIntensityAnalyzer, the majority of tweets have neutral sentiment. This demonstrates that a more nuanced study of the data is required to extract meaning.
https://www.nltk.org/api/nltk.sentiment.html
