# Supplementary material for: Understanding the vaccine stance of Italian tweets and addressing language changes through the COVID-19 pandemic: Development and validation of a machine learning model
Source: Front Public Health. 2022 Jul 29;10:948880. doi: 10.3389/fpubh.2022.948880 (PMC9372360; doi:10.3389/fpubh.2022.948880)
Supplement: Supplementary file 6 [file Image_5.PDF]

Limitations of Twitter data

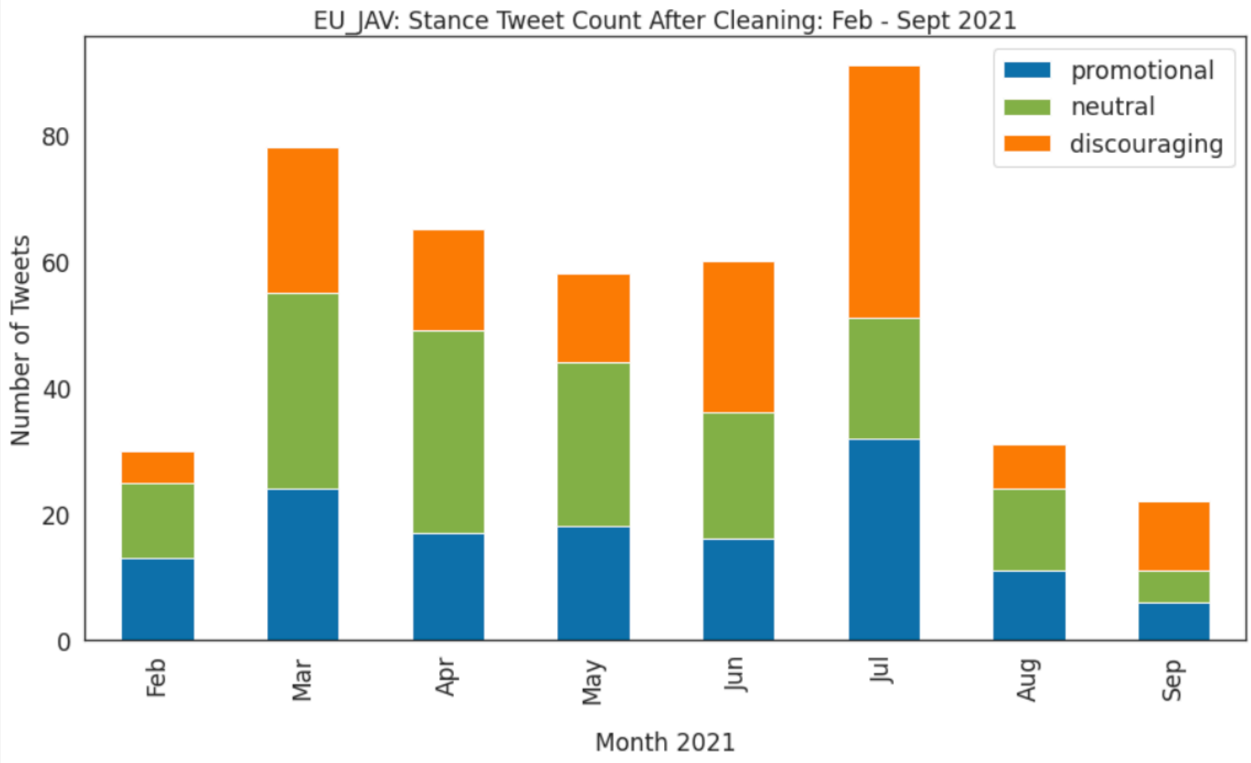

The number of tweets per month collected is not constant. Snap shots of the public stance are limited by data collection methods.
