# Supplementary material for: Understanding the vaccine stance of Italian tweets and addressing language changes through the COVID-19 pandemic: Development and validation of a machine learning model
Source: Front Public Health. 2022 Jul 29;10:948880. doi: 10.3389/fpubh.2022.948880 (PMC9372360; doi:10.3389/fpubh.2022.948880)
Supplement: Supplementary file 7 [file Table_1.PDF]

Machine learning terminology

Bidirectional Encoder Representations from Transformers (BERT)

**Bidirectional**	Text is not analysed from left-to-right or right-to-left but the entire sequence of words is read all at once. The context of the word can then
**Encoder**	The input sequence of words is mapped into an n-dimensional vector (A decoder converts vectors back into words). BERT-base has 12 encoder layers whilst BERT large has 24 encoder layers.
**Representations**	Vectors represent words in the sequence
**Transformer**	The transformer connects the encoder and decoder through an attention mechanism. It weighs the importance of different parts of the text thus identifies the context that confers meaning to each word in a sentence.

BERT models come pre-trained on huge unlabelled datasets which then can be fine-tuned with a much smaller task-specific dataset.

A hyperparameter is a parameter that is set before the machine learning process begins. These parameters are tuneable and can directly affect how well a model performs. The four key hyperparameters in this study were epochs, learning rate, warm-up and batch-size.

**Epochs**	In one epoch, all of the data is used exactly once to train the model. More training improves the model but overtraining can make the model too specific to the training data and reduce performance on new data.
**Learning rate**	Determines the step size at each iteration while moving towards minimising the difference between estimated and true values. In setting a learning rate, there is a trade-off between the rate of convergence and overshooting, like a moving ball settling into the bottom of a well.
**Warm-up**	Allows the model to gradually stabilise. Uses a percentage of the data to warm-up with a smaller learning rate, then increases the learning rate as
**Batch-size**	Bigger batches means more parallelisation and therefore less time to run computation, but can lead to poor generalisation of results.

Machine learning metrics to study the effects of tuning and training

**Accuracy**	Fraction of correct predictions.
**Precision**	The ability of the classifier not to label as true a sample that is false.
**Recall**	The ability of the classifier to find all the true samples.
**F-score**	Harmonic mean of precision and recall.

**Support**	The number of samples used for testing.
**Macro avg**	Unweighted mean for each label. This does not take label imbalance into
**Weighted avg**	Mean weighted by the number of true instances for each label. This alters ‘macro’ to account for label imbalance.

When training a model overfitting should be avoided

Overfitting	A model is trained on a finite set of training data. The model generalises so it can predict using unseen data. Overfitting occurs when a model fits the data in training well but incurs large error in generalisation.
Early stopping	A form of regularisation or smoothing used to avoid overfitting.
Loss	The penalty for a bad prediction: If the model's prediction is perfect, the loss is zero; otherwise, the loss is greater than zero.
