# Supplementary material for: Understanding the vaccine stance of Italian tweets and addressing language changes through the COVID-19 pandemic: Development and validation of a machine learning model
Source: Front Public Health. 2022 Jul 29;10:948880. doi: 10.3389/fpubh.2022.948880 (PMC9372360; doi:10.3389/fpubh.2022.948880)
Supplement: Supplementary file 8 [file Table_2.PDF]

Data cleaning steps and number of tweets remaining after each action

**Data Cleaning steps**	**dataset A** Number of tweets	**dataset B** Number of tweets	**dataset A+B** Total number of tweets
Downloaded tweets	3000	800	3800
Non relevant tweets removed	2473	676	3149
Replies removed	1729	529	2258
Retweets removed	1704	529	2333
Duplicates removed	1382	529	1911
Less than 5 words removed	1378	526	1904
